# Including endophenotypes as covariates in variance component heritability and linkage analysis

**DOI:** 10.1186/1471-2156-6-S1-S49

**Published:** 2005-12-30

**Authors:** Julia N Bailey

**Affiliations:** 1 Neuropsychiatric Institute and Epilepsy Genetics/Genomics Laboratories, Comprehensive Epilepsy Program, University of California at Los Angeles School of Medicine, Los Angeles, CA, USA and Veteran's Administration Greater Los Angeles Healthcare System-West Los Angeles, CA, USA

## Abstract

The purpose of these analyses was to determine if incorporating or adjusting for covariates in genetic analyses helped or hindered in genetic analyses, specifically heritability and linkage analyses. To study this question, two types of covariate models were used in the simulated Genetic Analysis Workshop 14 dataset in which the true gene locations are known. All four populations of one replicate were combined for the analyses. The first model included typical covariates of sex and cohort (population) and the second included the typical covariates and also those related endophenotypes that are thought to be associated with the trait (phenotypes A, B, C, D, E, F, G, H, I, J, K, and L). A final best fit model produced in the heritability analyses was used for linkage. Linkage for disease genes D1, D3, and D4 were localized using models with and without the covariates. The use of inclusion of covariates did not appear to have any consistent advantage or disadvantage for the different phenotypes in regards to gene localization or false positive rate.

## Background

The analyses of complex traits can be complex. Often the phenotype itself is not defined or well measured, and voluminous information is collected that relates to the trait. While this increased phenotyping can be helpful in analyses, there still remains a question as to the best methods for incorporating additional information into the genetic analyses: "How do we analyze all the data together?"

The Genetic Analysis Workshop 14 (GAW14) dataset was simulated to reflect realistic issues in study design and data collection. Data were gathered from several different research groups using different ascertainment schemes and different affection criteria. The data therefore are heterogeneous, reflecting reality. The purpose of these analyses was to determine if the inclusion of typical covariates (sex and population or cohort) and endophenotype traits (phenotypes A, B, C, D, E, F, G, H, I, J, K, and L) improved the genetic analyses of Kofendrerd Personality Disorder (KPD) and the 12 endophenotypes.

## Methods

To imitate a realistic situation, only one replicate (23) of the simulated GAW14 dataset was used. All families from all four populations were included, even though each population had different ascertainment schemes. The affection status for KPD and all phenotypes (A, B, C, D, E, F, G, H, I, J, K, and L) were studied. Each phenotype was analyzed with 3 types of variance component models: 1) without any covariates included in the model, 2) with sex and population (if they were significant, otherwise this model was not performed), 3) with significant variables of sex, population, and the other 12 endophenotype traits. Each covariate was tested independently for significance, and only significant covariates (*p *< 0.05) were included in the final model.

Heritability and linkage analyses were performed using variance component analyses or random effects models [1-4] as implemented in the computer program SOLAR [4]. The variance component method [2] decomposes the phenotypic variation (Ω) into measured (candidate gene) genetic effects (Πσ_m_^2^), unmeasured genetic effects (2φσ_g_^2^), and other effects (Iσ_e_^2^). Ω = Πσ_m_^2 ^+ 2φσ_g_^2 ^+ Iσ_e_^2^, where σ_m_^2 ^is the additive genetic variance due to the major locus, and Π is a matrix of elements that provide the probability that individuals i and j are identical-by-descent (IBD) at a trait locus that is linked to a genetic marker locus. Π is a function of the estimated IBD matrix of the genetic marker itself and a matrix of the correlations between the proportion of genes IBD at the marker and at the trait. σ_g_^2 ^is the genetic variance due to residual additive genetic factors, φ is the kinship matrix, σ_e_^2 ^is the variance due to individual-specific environmental effects, and I is an identity matrix. The dichotomous variables were analyzed modeling the discrete affection status trait as a threshold model [5], whereas the latent liability is assumed to have an underlying multivariate normal distribution. Covariates can be added to the model and their effects are estimated simultaneously with the variance components by maximum likelihood techniques. Likelihood ratio tests were performed to test for heritability and locus effects, where the likelihood of the model is compared to a restricted model with no linkage. Twice the difference in log likelihood of the variance component models yields a test statistic that is asymptotically distributed as a 1/2:1/2 mixture of a χ^2 ^variable and a point mass at zero. Two point linkage analyses were performed using all of the genome scan markers. LOD scores > 3.3 were considered significant for linkage from the genome scans [6].

## Results and Discussion

The inclusion of the endophenotypes as covariates into the variance component models influenced some of the heritability estimates and linkage analyses.

### Heritability

Not all of the phenotypic traits were heritable (Table 1). KPD was not heritable when analyzed without covariates, or when analyzed with just sex and population as covariates. When the associated phenotypes were tested, seven of the 12 were significant. When these significant phenotypes were included in the model KPD heritability went to 1.0 and became very significant (*p *< 0.000001). It appears that there may be a significant genetic component to KPD that is not explained by the associated phenotypes, however the heritability parameter is at a boundary that may indicate instability.

Five of the phenotypes appear to have a significant genetic component either with or without covariates: phenotypes A, B, D, K, and L. Interestingly, phenotypes C and G appeared heritable when analyzed without any covariates, but the heritability is no longer significant when the associated phenotypes are included in the model.

Surprisingly, population was not as much of a confounder as expected; it was only significant for phenotype D. Population was associated with KPD when only population and sex were included in the model. Sex was significant for KPD, phenotype I, and phenotype L.

### Linkage

The inclusion of covariates in the linkage analyses did not produce consistent results among the phenotype variables as to the localization of genes (Tables 2 and 3). Diseases genes D1, D3, and D4 would have been localized in both scenarios, and gave no evidence at the other locations.

Forty loci were significant for KPD with the inclusion of covariates, however none of them was the closest to the disease gene. These markers with LODs are listed, along with the LODs near the true disease locations in centimorgans: D01S0011 (3.68), D01S0016 (4.15), D01S0017 (5.07), D01S0018 (3.61), D01S0023 *D1* (2.80), D01S0034 (4.24), D02S0043 *D6* (3.25), D02S0057 (4.62), D02S0075 (3.36), D02S0076 (4.50), D02S0080 (3.94), D02S0081 (3.78), D02S0082 (3.42), D03S0126 (4.06), D04S0128 *D2* (0.00), D04S0145 (3.83), D04S0159 (4.40), D04S0160 (3.79), D04S0171 (4.12), D05S0172 *D3* (1.54), D05S0174 (3.84), D05S0183 (3.49), D05S0211 (3.37), D05S0213 (3.38), D06S0222 (3.86), D06S0229 (4.11), D06S0232 (3.50), D06S0244 (3.92), D06S0246 (4.83), D06S0248 (3.95), D07S0264 (3.78), D07S0271 (3.61), D07S0274 (4.69), D07S0276 (3.82), D07S0289 (4.06), D08S0304 (3.77), D08S0329 (4.32), D09S347 *D4* (1.19), D09S0350 (3.71), D09S0356 (3.83), D09S0376 (3.53), D09S0381 (4.41), D09S0388 (4.23), D10S0400 *D5* (0.47), D10S0413 (4.06), and D10S0414 (3.46). These false positives could be due to violations of assumptions about the distribution of the trait; specifically the assumption that there is a threshold and an underlying distribution. The simulation parameters show that the assumption is violated as unaffecteds and affecteds have different genetic liabilities. This could explain why heritability is unstable and goes to a boundary when adjusted for covariates.

Disease genes D1, D3, and D4 for phenotypes A, B, and K were localized with or without the inclusion of covariates. Genes were only localized for Phenotypes C, D, G, and L with models that did not include the covariates.

Heritability was no longer significant for phenotype C or G when covariates were in the model. The use of endophenotypes for covariates may have regressed out the major gene effects. Therefore, any genes revealed in the analyses without the covariates should reveal shared effects. Phenotypes C and G showed linkage to D3 and D4, genes identified in analyses of other phenotypes.

Several false positives were found in the search for disease genes, including a false-positive region quite distal to any disease genes. The inclusion of covariates did not consistently alter the false-positive rates.

## Conclusion

Simulated disease genes D1, D3, and D4 were localized by variance component linkage analyses to the endophenotypes of the simulated KPD trait. Adjusting models for covariates and the other endophenotypes did not consistently alter the findings.

## Abbreviations

GAW14: Genetics Analysis Workshop 14

IBD: Identical by descent

KPD: Kofendrerd Personality Disorder

## Figures and Tables

**Table 1:** Heritabilities of KPD affection status and associated phenotypes with and without the inclusion of covariates (covs)

	h^2 ^no covs	h^2 ^with covs	pop	sex	A	B	C	D	E	F	G	H	I	J	K	L
KPD	0.5	1.00**	ns^a^	*	ns	**	*	**	**	**	*	**	ns	ns	ns	ns
A	1.0**	1.00**	ns	ns	-	**	ns	ns	ns	ns	ns	ns	ns	ns	*	ns
B	0.31**	0.62**	ns	ns	**	-	ns	*	**	**	**	**	ns	ns	**	ns
C	0.21**	0.11	ns	ns	ns	*	-	**	ns	*	**	**	ns	ns	ns	ns
D	0.26**	0.25**	*	ns	ns	**	**	-	**	**	**	**	ns	ns	ns	ns
E	0.01	0.00	ns	ns	ns	**	*	**	-	**	**	**	ns	ns	ns	ns
F	0.03	0.11	ns	ns	*	**	*	**	**	-	**	**	ns	ns	**	ns
G	0.23**	0.07	ns	ns	ns	**	**	**	*	*	-	ns	ns	ns	**	**
H	0.01	0.10	ns	ns	ns	**	**	**	**	**	*	-	ns	ns	**	ns
I	0.00	0.00	ns	*	ns	ns	ns	ns	ns	ns	*	ns	-	ns	ns	ns
J	0.07	0.07	ns	ns	ns	ns	ns	ns	ns	ns	ns	ns	ns	-	ns	ns
K	0.58**	0.60**	ns	ns	ns	**	ns	*	ns	**	*	**	ns	ns	-	**
L	0.90**	0.95**	ns	*	ns	*	ns	ns	ns	ns	*	ns	ns	ns	*	-

*p < 0.05

** p < 0.0001

^a^ns, indicates non-significant tests.

**Table 2:** All significant (>3.3) LOD scores for the endophenotypes analyzed without covariates

		Phenotype					
		
		A	B	C	D	G	K
D01S0017			3.9				
D0S0018			4.3				
D0S019			6.2				
D0S020			7.5				
D0S021			5.7				
D01S0022		5.2	11.7				
D01S0023^a^	D1	9.0	11.5				
D01S0024		3.6	11.4				
D01S0025		3.5	12.2				
D01S0026			8.7				
D01S0027		6.7	12.0				
D01S028			3.7				
D01S029			4.5				
D01S038							5.3
D03S0116		3.6					
D03S0123							5.6
D03S0124							4.2
D03S0125							3.6
D03S0126							6.0
D03S0127							16.5
D10S0172	D3			3.7	6.1		
D05S0173		6.7			4.7		
D09S0347	D4			4.8		3.6	4.8
D09S0348							

**Table 3:** All significant (>3.3) LOD scores for the endophenotypes analyzed with covariates

		A	B	K
D01S0022			3.8	
D01S0023^a^	D1	3.8		
D01S0038				4.3
D03S0116		5.0		
D03S0123				4.3
D03S0124				3.5
D03S0127				6.6
D05S0172	D3	8.6		
D05S0173		11.6		
D05S0174		7.3		
D06S0259		4.7		
D07S0287		3.4		
D09S0347	D4			12.2
D09S0348				7.6
D09S0350				4.0
